# Physical exercise for primary sarcopenia: an expert opinion

**DOI:** 10.3389/fresc.2025.1538336

**Published:** 2025-03-28

**Authors:** Antimo Moretti, Federica Tomaino, Marco Paoletta, Sara Liguori, Silvia Migliaccio, Mariangela Rondanelli, Angelo Di Iorio, Raffaello Pellegrino, Davide Donnarumma, Daniele Di Nunzio, Giuseppe Toro, Francesca Gimigliano, Maria Luisa Brandi, Giovanni Iolascon

**Affiliations:** ^1^ Department of Medical and Surgical Specialties and Dentistry, University of Campania “Luigi Vanvitelli”, Naples, Italy; ^2^ Department of Mental and Physical Health and Preventive Medicine, University of Campania “Luigi Vanvitelli”, Naples, Italy; ^3^ Department of Experimental Medicine, University Sapienza of Rome, Rome, Italy; ^4^ Department of Public Health, Experimental and Forensic Medicine, University of Pavia, Pavia, Italy; ^5^ Laboratory of Clinical Epidemiology, Department of Medicine and Sciences of Aging, University G. D'Annunzio, Chieti, Italy; ^6^ Department of Scientific Research, Campus Ludes, Off-Campus Semmelweis University, Lugano-Pazzallo, Switzerland.; ^7^ Rehabilitation Unit, University Hospital ‘Luigi Vanvitelli’, Naples, Italy; ^8^ Donatello Bone Clinic, Villa Donatello Hospital, Sesto Fiorentino, Italy

**Keywords:** sarcopenia, exercise, resistance training, aerobic training, balance training, multimodal exercise

## Abstract

Sarcopenia is the age-related loss of skeletal muscle mass and function. Recently, research has focused on defining diagnostic criteria for this condition, now recognized as a muscle disease with a specific identifying code (ICD-10: M62.84). The diagnostic process for sarcopenia involves several stages, including the use of dedicated questionnaires and objective measurements of muscle strength and mass. According to international guidelines, therapeutic exercise is recommended to improve muscle mass, muscle strength, and physical performance. However, much of the supporting evidence comes from studies on non-sarcopenic elderly patients. Among types of therapeutic exercise, guidelines mainly emphasize muscle strengthening. The prescription of therapeutic exercise must consider the clinical and functional conditions of the patient (e.g., the presence of severe sarcopenia) and patient preferences. Muscle strengthening should target large muscle groups and include low-intensity resistance exercise for strength improvement, or high-intensity resistance exercise for additional benefits in muscle mass and function. Evidence suggests that an ideal therapeutic exercise program for sarcopenic patients should be multimodal, incorporating muscle strengthening, aerobic exercise, and balance control programs. This approach could enhance patient adherence by offering variety. Although multimodal therapeutic exercise improves muscle mass and function, these benefits can be lost during prolonged physical inactivity. Therefore, the exercise prescription must define intensity, volume (repetitions and sets), frequency, rest intervals, and duration, tailored to the type of exercise. Aerobic training programs improve endurance and optimize mitochondrial function. Balance training, important for reducing the risk of falls, should be done at least three times a week. Muscle strengthening should be done at least two days a week, starting at 50%–60% of 1 repetition maximum (RM) and progressing to 60%–80% of 1 RM, with approximately 10 exercises per session. Adopting comprehensive prescription protocols, such as those proposed in this paper, can significantly aid in the functional recovery and well-being of patients with sarcopenia.

## 1 Introduction

Sarcopenia is a degenerative and progressive disorder of skeletal muscle characterized by a reduction in muscle mass and function (1). This age-related disease has significant consequences, including an increased risk of falls, mobility limitations, decreased independence in daily activities, and higher mortality rates (2). At socio-economic level, the estimated cost of hospitalization for people with sarcopenia was very high, reaching over USD $40 billion (3). This increased economic burden is due to greater odds of hospitalization and on average more hospital stays in sarcopenic individuals compared to people without sarcopenia.

Despite these impacts, identifying sarcopenia is challenging due to the lack of a universal operational definition. However, a case-finding strategy might be effective, as suggested by both the European Working Group on Sarcopenia in Older People 2 (EWGSOP2) (1) and the Asian Working Group for Sarcopenia (AWGS) (4). Both groups proposed using the SARC-F questionnaire as a screening tool (1, 5). If a patient scores higher than 4 on the SARC-F, further assessment of muscle strength using a handheld dynamometer is required (6). An alternative test for muscle strength assessment is the sit-to-stand test, which measures the time taken to stand up and sit down five times consecutively without using the upper limbs (7). To confirm a diagnosis of sarcopenia, it is essential to measure muscle mass using dual-energy x-ray absorptiometry (DXA) or bioelectrical impedance analysis (BIA) (8, 9). Additionally, assessing the severity of sarcopenia involves testing physical performance with specific tools, such as the 4-meter walking speed, the Short Physical Performance Battery (10), the Time Up and Go test, or the 400-meter walk test (11). Table 1 summarizes the diagnostic cut-offs for each parameter according to the EWGSOP2 and the AWGS recommendations.

**Table 1:** Key indicators used for the diagnosis of sarcopenia according to European and Asian working groups on sarcopenia.

Parameter	EWSGOP2 threshold value	AWGS threshold value
SARC-F	≥4	≥4
SARC-calf	N/A	≥11
Calf circumference	N/A	Men: <34 cm, Women: <33 cm
Hand grip strength	Men: <27 kg, Women: <16 kg	Men: <28 kg, Women: <18 kg
Chair stand test	≥15 s for 5 repetitions	≥12 s for 5 repetitions
ASM	By DXA or BIA: Men: <20 Kg, Women: <15 Kg	N/A
ASM/h^2^	By DXA or BIA: Men: <7 Kg/m^2^ Women: <5.5 Kg/m^2^	By DXA: Men: <7 Kg/m^2^ Women: <5.4 Kg/m^2^ By BIA: Men: <7 Kg/m^2^ Women: <5.7 Kg/m^2^
SPPB	≤8	≤9
Gait speed	≤0.8 m/s on 4 meters	<1 m/s on 6 m
TUG	≥20 s	N/A
400 m walk test	≥6 min	N/A

ASM, appendicular skeletal muscle mass; SPPB, short physical performance battery; TUG, time up and go.

The appropriate management of sarcopenic patients encompasses nutritional and physical exercise interventions (12–14). To date, current evidence tends to recommend a higher intake doses of key nutrients, including protein (25–30 g per meal), leucine (77.8 mg/kg/day for men and 78.2 mg/kg/day for woman), and vitamins (D, C, and E), among other essential elements (15, 16). In the future, complex hybrid nutritional supplements will be developed to personalize the nutritional experience based on the metabolic status of each individual patient (17). Currently, no specific pharmacological treatments for sarcopenia have been approved by international regulatory agencies (18), making exercise the frontline treatment against age-related muscle wasting (19). Physical exercise stimulates the production of various myokines that have anti-inflammatory and anabolic effects (20). It activates specific pathways, including the activation of adenosine monophosphate-activated protein kinase (AMPK), which enhances myofiber hypertrophy, mitochondrial biogenesis, and angiogenesis (21). Additionally, regular progressive resistance and aerobic exercise promotes reduction of adipose tissue improving insulin sensitivity and glucose metabolism. Furthermore, physical activity has cognitive benefits, such as increasing cerebral flow and elevating levels of neurotrophic factors like BDNF and insulin-like growth factor-1 (IGF-1), while decreasing neurotoxic factors such as C-reactive protein, cortisol, and interleukin-6 (IL-6), as well as modulating other inflammatory cytokines (22).

The need for this expert opinion also arises from the increased interest for this condition following its inclusion in the International Classification of Diseases as a muscle pathology (ICD-10: M62.84) (23, 24).

Current guidelines strongly recommend exercise for managing sarcopenia (14). However, the studies included in this guideline did not involve patients diagnosed with sarcopenia, according to the subsequently published EWSGOP2 and AWGS criteria, limiting their applicability. This manuscript aims to address this knowledge gap by emphasizing the need for tailored physical exercise guidelines for people affected by primary sarcopenia and providing recommendations for their implementation in clinical practice.

## 2 Materials and methods

We collected and assessed scientific evidence on physical exercise in sarcopenia. We considered for the search strategy studies including patients with diagnosis of sarcopenia according to the criteria of the EWGSOP2 or the AWGS. The included studies were randomized controlled trials (RCTs), systematic review and meta-analyses of RCTs, addressing the efficacy of physical exercise for sarcopenia compared to other interventions, such as nutritional approaches, or no intervention. The primary outcomes were muscle mass, strength, physical performance, and risk of falls. The literature search was conducted via the PubMed database from inception until December 31st, 2023. The following set of MeSH terms was used: “Sarcopenia”, “Exercise” and ‘Resistance training’. Two independent reviewers (M.P. and S.L.) evaluated abstracts and full texts based on established inclusion criteria.

## 3 The role of physical exercise on primary sarcopenia: evidence synthesis

Most studies suggest prescribing resistance exercise (RE) alone or in combination with other exercise modalities. Undoubtedly, RE is a cornerstone intervention in managing sarcopenia, although proposed protocols vary widely among the studies.

In a systematic umbrella review, (14 studies, 7 of which conducted a meta-analysis), Beckwée and al (25). suggest that low-load resistance training (LIRT) with intensity up to 50% of one-repetition maximum (1RM) improves muscle strength. More in detail, LIRT is primarily focused on building endurance and improving muscle efficiency, making it suitable for activities requiring sustained effort over long periods. Conversely, high-intensity resistance training (HIRT) at 80% of 1RM maximizes strength gains. The HIRT is geared toward maximizing muscle strength and size, leveraging the benefits of heavier loads and lower repetitions. Depending on an individual's fitness goals, choosing between LIRT and HIRT can significantly influence the physiological outcomes and overall training effectiveness. Regarding volume, frequency, and duration, authors recommend performing 1–4 sets of 8–15 repetitions, 2–3 times per week, over 6–12 weeks. Evidence also supports multimodal exercise, combining RE, aerobic training, and balance exercises, as well as blood flow restriction (BFR) training. Balance training involves exercises designed to improve stability and coordination, yielding significant biological effects across various body systems. This training enhances neuromuscular control by improving communication between the nervous system and muscles, which boosts proprioception, coordination, and reaction times, thereby reducing fall risk. Moreover, balance training engages multiple muscle groups, strengthening stabilizer muscles and promoting overall muscular development and endurance (22).

BFR is a technique that creates a controlled environment in which venous blood flow is restricted while maintaining arterial blood flow to the targeted muscles. This method allows individuals to achieve significant muscle adaptations with lighter weights, reducing injury risk and minimizing stress on joints. By restricting venous outflow, BFR training promotes metabolic buildup in muscles, through a localized metabolic accumulation in muscle tissue, creating a hypoxic environment that stimulates hypertrophic signaling pathways. The hypoxic environment in BFR training promotes metabolite buildup, such as lactate, which enhances growth hormone production and fast-twitch fiber recruitment. These effects, driven by mTORC1 and MAPK pathways, facilitate hypertrophy and strength gains even at low intensities (≥20% 1RM), making low-load BFR a safer alternative to high-load resistance training (26, 27). Compared to LIRT, BFR appears more effective for enhancing muscle strength at low intensity but remains training less effective than HIRT.

Among RE modalities, kettlebell training (KT) stands out for its dynamic nature and full body engagement (28), making it suitable for older patients (29). Chen HT et al. (29) studied 33 elderly women with sarcopenia (65–75 years) divided into a KT group and a control group (CON). The KT group followed an 8-week training program, while the CON group maintained their usual lifestyle. At 8 and 12 weeks, the KT group demonstrated significant improvements in muscle mass, sarcopenia index, grip strength, back strength, and peak expiratory flow (PEF), with effects persisting at 4 weeks. Serum high-sensitivity C-reactive protein (hs-CRP) levels were also significantly lower in the KT group. The KT protocol involved kettlebells at 60%–70% of 1RM, with 11 movements targeting major muscle groups, incorporating chairs for safety. Progression ranged from basic to advanced exercises, with resistance adjusted based on individual capacity. Resistance was adjusted based on individual physical capacity, providing gradual increases when repetitions exceeded 10 and decreased when they fell below 8. Load and planning changed every two weeks, involving both upper and lower limbs. The frequency of workouts included a 48-hour interval between sessions. This approach enhances safety, inclusivity, and compliance in sarcopenic individuals. Despite all these benefits, exercise selection should consider individual limitations, and while KT offers dynamic and full-body engagement, its suitability depends on the individual's ability to safely perform the activity.

In osteosarcopenia, a condition characterized by the concurrent presence of both sarcopenia and osteopenia/osteoporosis, RE appears both feasible and safe. The FrOST study, a one-year RCT involving 43 elderly men (73–91 years), examined the effects of twice-weekly high-intensity RE combined with whey protein, vitamin D, and calcium supplementation (30). Resistance training is structured into four progressive phases, each increasing in difficulty. It includes a high-intensity dynamic resistance training (DRT) regimen that emphasizes single-set training at high intensity and effort, targeting both major and minor muscle groups. The exercise intensity is organized around specific repetition ranges (5–7 or 8–10) and effort levels, defined as work to failure (non-repetition maximum, nRM). The exercise group showed significant preservation of lumbar spine bone mineral density (BMD), increased skeletal muscle mass index (SMI), and improved hip extensor strength. In contrast, the control group experienced significant reduction in spine BMD and SMI, underscoring the detrimental effects of neglecting high intensity RE in osteosarcopenia management. Given the impact of exercise mode and selection on bone adaptations, variations in movement patterns, loading strategies, and multi-directional exercises should be integrated to optimize skeletal benefits. For optimal results in improving bone mineral density (BMD) at the neck of the femur, as reported by Benedetti et al. (31), it is recommended to engage in progressive resistance exercise for the lower limbs at least three times a week over the course of a year. This type of strength training effectively increases bone density at specific sites, particularly the neck of the femur and lumbar spine, with benefits sustained in the short to medium term.

The meta-analysis conducted by Shen et al. (32) highlights the efficacy of RE alone or combined with aerobic exercise and balance training (BT), in improving quality of life in elderly patients with sarcopenia. Combined nutritional and exercise interventions significantly improve grip strength compared to exercise alone. Additionally, combining RE with BT is the most effective approach for enhancing physical performance.

Liang et (33). further explored this in a single-blind RCT involving very elderly individuals (80–99 years). A 12-week program combining BT and RE significantly improved functional independence, as assessed by the Barthel Index, though it did not significantly reduce fall incidence.

Aerobic exercise complements RE by enhancing mitochondrial function, muscle endurance and cardiovascular health. For sarcopenic patients, a recommended regimen includes 30 min per day, three or more times per week, for at least five months. Progression is essential, starting with low intensity exercise (40% of maximum heart rate) and advancing to moderate (50%–60%) and high-intensity (>60% of maximum heart rate) phases over time. Activities such as walking, jogging, cycling, swimming, dancing, and tai chi are commonly recommended (34–37).

In the meta-analysis of 42 RCTs (3,728 elderly participants) conducted by Shen et al. (32), aerobic exercise alone or with nutritional interventions improved quality of life compared to non-exercise interventions. Adding balance or aerobic training to RE is particularly effective for physical performance improvement.

When comparing nutritional interventions plus RE to RE alone, Tokuda et al. demonstrated that supplementation with essential amino acids (EAA) and tea catechins (TCC) following RE may enhance skeletal muscle mass (SMM) in older adults with sarcopenia. However, supplementation with EAA alone after RE did not provide additional benefits beyond RE alone (38).

The molecular mechanisms underlying exercise benefits remain under investigation. Robinson et al. (37) demonstrated that high-intensity interval training (HIIT) improves insulin sensitivity, skeletal muscle mitochondrial respiration, lean mass, and aerobic capacity in both young and elderly individuals. HIIT induces greater increases in gene transcripts, particularly in mitochondrial proteins, than other training modes, suggesting its potential to reverse age-related mitochondrial decline. These adaptations include enhanced mitochondrial biogenesis, improved muscle mass and function.

Physical activity offers extramuscular benefits through ‘exerkins’, signaling molecules released in response to muscle contractions (39). These molecules, such as angiopoietin-1, FGF21, and IL-6 play autocrine, paracrine, and endocrine roles, influencing cardiovascular, metabolic, immune, and neurological health. These findings highlight exercise’ holistic benefits, making it indispensable in sarcopenia management.

**Figure 1:**
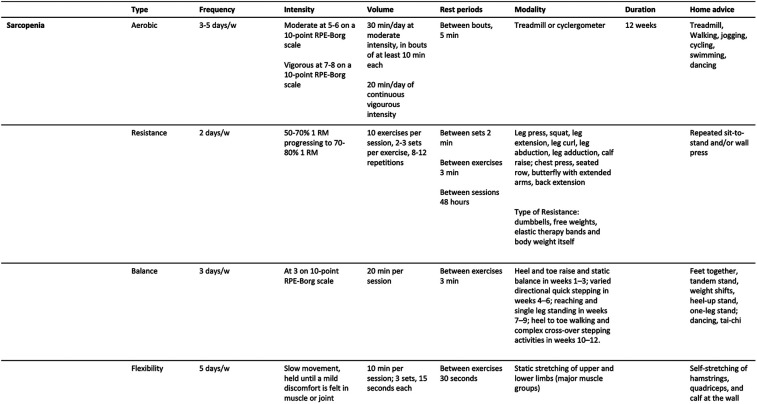
Practical recommendations for therapeutic exercise prescription for people with primary sarcopenia.

**Figure 2:**
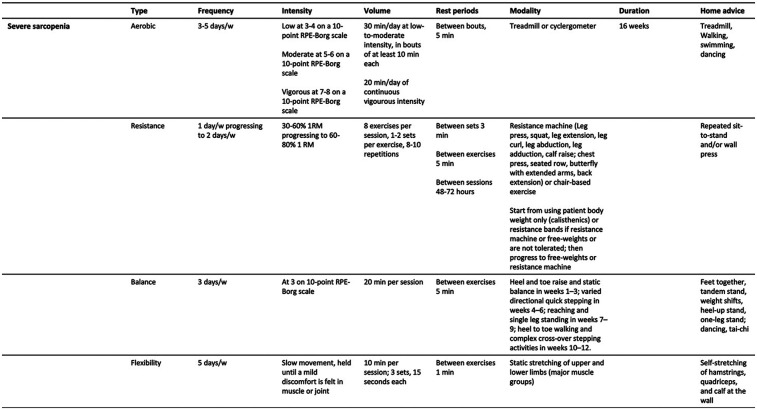
Practical recommendations for therapeutic exercise prescription for people with primary sarcopenia (severe sarcopenia).

**Figure 3:**
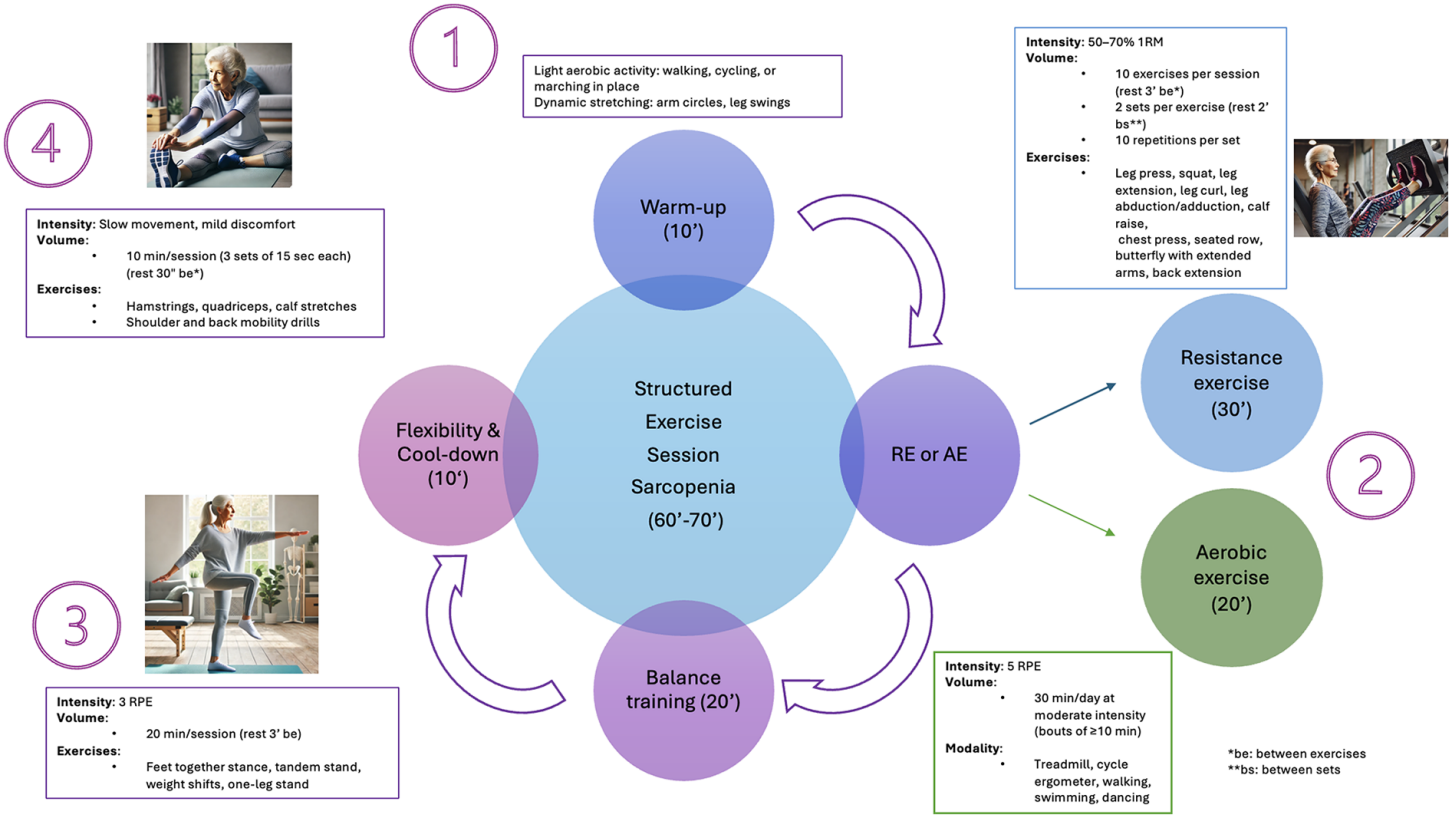
A proposed structured exercise session for patients with primary sarcopenia.

**Figure 4:**
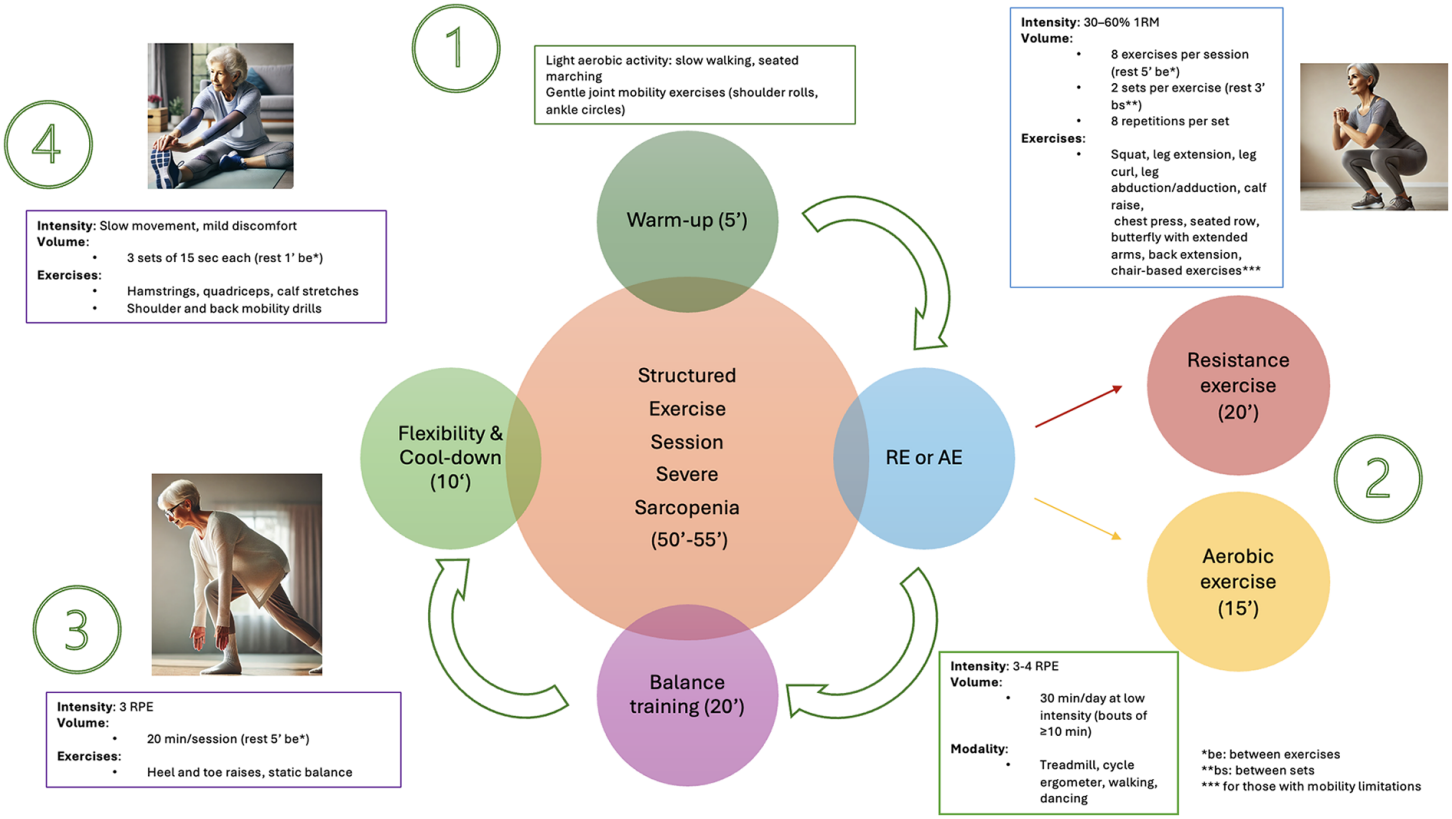
A proposed structured exercise session for patients with severe sarcopenia.

Practical recommendations for managing primary sarcopenia and severe sarcopenia are summarized in Figures 1, 2. A proposed session, tailored for patients with primary sarcopenia and severe sarcopenia, is presented in Figures 3, 4.

## 4 Clinical applications

These recommendations provide a comprehensive framework for translating scientific advancements into clinical practice. When prescribing therapeutic exercise, it is crucial to personalize the regimen based on individual needs and specific patient conditions. Baseline functional assessments, along the EWGSOP2/AWGS criteria form the foundations for targeted prescriptions. To identify and assess the severity of sarcopenia, various tools can be employed, such as the SARC-F questionnaire, muscle strength measurements like the handgrip strength test, and other evaluations, including walking speed and the SPPB. The Society on Sarcopenia, Cachexia and Wasting Disorders (SCWD) emphasizes tailoring exercise programs to align with patient's goals and preferences to improve adherence, since particularly give the commonly low levels of physical activity among the elderly (40). This approach is particularly crucial when considering the commonly low levels of physical activity among the elderly population. By customizing exercise plans to reflect the unique interests, motivations, and physical capabilities of older adults, healthcare providers can foster a greater sense of ownership and commitment to their physical activities program. Strategies to increase physical activity participation among older adults should focus on raising awareness of the benefits while also addressing and minimizing the perceived risks associated with physical activity (41). Finally, providing a video and brochure about the exercise, as well as using phone or email reminders, could improve the integration of certain activities at home. On the other side, when it is possible, patient adherence can be significantly improved by fostering social interaction and support through organized group exercise sessions, that actively involve caregivers, family members, and friends. These collaborative activities not only create a sense of community and belonging but also encourage individuals to remain committed to their health and fitness goals.

Factors such as intensity, volume, and progression should be carefully considered. Resistance exercise should play a key role in the prescription, serving as both as a preventive and therapeutic intervention. The most important training principles include progressive overload, specificity, individualization, and periodization. Considering the different sub-types of RE available (i.e., traditional, cluster-set RE, suspension, high-speed RE, etc), low-load power-based training, which emphasizes faster movement execution and shorter rest periods between sets, can be particularly beneficial, especially in cases of advanced sarcopenia (42).

However, it is important to note that while physical exercise provides significant benefits in managing sarcopenia, excessive exercise raises concerns regarding the relationship among volume, intensity, and cardiovascular risks. Careful evaluation of the balance between exercise quantity and intensity is essential, especially when considering potential cardiovascular complications. These complications may include accelerated coronary artery calcification, myocardial fibrosis, atrial fibrillation, and an increased risk of sudden cardiac death, particularly in the elderly (43).

A major challenge in implementing exercise interventions for older adults and sarcopenic patients is limited compliance, often due to personal, family, or occupational commitments. Consequently, evidence of maintaining muscle health after exercise training periods is essential. A recent narrative review suggests that muscle strength and size can be preserved for up to 32 weeks with as few as 2 sessions per week and 2–3 sets per exercise, provided exercise intensity is maintained (44). While most evidence supports muscle strengthening for managing sarcopenia, the optimal type of RE remains inadequately defined, particularly concerning isometric vs. dynamic contractions. This represents an unmet need, as different types of muscle actions may yield distinct outcomes in terms of both effectiveness and safety. For example, older adults participating in an eccentric training program not only demonstrated greater preservation of exercise-induced muscular adaptations compared with other training modalities, but also maintained power and strength for up to 3 months of detraining. These findings have significant implications for managing sarcopenia (45).

The diversity of physical abilities in the elderly population with sarcopenia requires individualized intervention strategies. For instance, for frail patients who struggle with conventional physical exercises, vibrational therapy has emerged as a promising alternative, as highlighted by a systematic review and meta-analysis (46). Vibration utilizes mechanical oscillations to improving muscle function by enhancing excitatory signaling from muscle spindles while reducing the inhibitory response from the Golgi tendon organ to the motoneuron pool (47). The mechanical stimulus generated is believed to engage proprioceptive spinal reflexes, thereby VT can be directed at specific muscles through two primary approaches: whole-body vibration, where participants either squat or stand on vibrating platforms, and local vibration, which is applied superficially to the targeted muscle (48).

## 5 Conclusions

Sarcopenia, a significant cause of disability in the elderly, requires early diagnosis and comprehensive intervention. Accurate assessment, beginning as early as age 50, based on mobility, muscle strength, and body composition, is crucial. Alongside nutritional strategies, therapeutic exercise emerges as a cornerstone of effective management. Multimodal exercise, which incorporates various exercise modalities, is preferable to single-modality programs. This preference is driven by the fact that single-modality exercises can more easily lead to muscle fatigue, be less enjoyable, and present challenges in maintaining long-term adherence. In contrast, a multimodal approach offers variety, making the training experience more enjoyable and sustainable over time (40). While RE remains an indispensable intervention to counteract the gradual loss of muscle mass, strength, and performance characteristic of severe sarcopenia, the multimodal exercise approach appears to be the most effective and suitable strategy for managing this condition. These recommendations, in addition to outlining practical prescription principles, are firmly rooted in evidence-based medicine (EBM).

In conclusion, early diagnosis and the adoption of an integrated approach from the earliest signs are imperative for managing sarcopenia. This approach underscores the essential synergy among clinical assessments, advanced diagnostic tools, and personalized interventions. Only through such a comprehensive strategy can we hope to counteract the development and the progression of primary sarcopenia, thereby reducing associated disability and mortality.
